# Regulatory mechanisms of GCN5 in osteogenic differentiation of MSCs in periodontitis

**DOI:** 10.1002/cre2.695

**Published:** 2023-04-06

**Authors:** Wei Lu, Li Zhang, Kun Ji, Ling Ding, Guofeng Wu

**Affiliations:** ^1^ Department of Prosthodontics, Nanjing Stomatological Hospital Medical School of Nanjing University Nanjing People's Republic of China; ^2^ Department of Pediatric Dentistry, Nanjing Stomatological Hospital Medical School of Nanjing University Nanjing People's Republic of China

**Keywords:** General control non‐repressed protein5 (GCN5), mesenchymal stem cells (MSCs), osteogenic differentiation, periodontitis

## Abstract

**Objectives:**

The regulatory mechanisms of GCN5 (General control non‐repressed protein5) in the osteogenic differentiation of mesenchymal stem cells (MSCs) in periodontitis are still unclear. The purpose of this review focuses on the regulating roles of GCN5 in bone metabolism and periodontitis, discusses the potential molecular mechanism and provides targets and new ideas for the treatment of periodontitis.

**Material and Methods:**

The integrative review methodology was used. Data sources include PubMed, Cochrane Library, and additional sources.

**Results:**

MSCs play an important role in the osteogenesis balance of periodontal tissue. Periodontal ligament stem cells (PDLSCs) from periodontitis patients exhibited defective osteogenic differentiation capacities. Histone acetylation is important in regulating the differentiation of different types of MSCs cells and is closely related to the reduced osteogenic differentiation of PDLSCs. GCN5, one of the first histone acetyltransferase linked to gene transcriptional activation, participates in many biological processes of mesenchymal stem cells. Downregulation of GCN5 expression and lack of GCN5 caused decreased osteogenic differentiation of PDLSCs. Intercellular information exchange may be an important way for MSCs to exert their regulatory and therapeutic functions.

**Conclusions:**

GCN5 affects the function of cell metabolism‐related genes by regulating the acetylation status of histones or non‐histones, thereby regulating some important progress of MSCs such as PDLSCs' osteogenic differentiation and BMCS osteogenic differentiation.

## 1 INTRODUCTION

Periodontitis is a kind of chronic inflammatory disease characterized by inflammation of the periodontal tissue and resorption of the alveolar bone. Periodontitis has been the primary cause of tooth loss because of its ability to cause continuous and irreversible destruction of periodontal tissue (Engebretson et al., 2013). Moreover, periodontitis can be associated with systemic diseases such as cardiovascular disease and diabetes mellitus thus affecting oral and general health (Katz et al., 2001). Conventional periodontal therapy is aimed at controlling inflammation and cannot regenerate or restore the function of periodontal tissues. Guided tissue regeneration and implantation of materials show limited ability in periodontal tissue repair (Chen et al., 2009; Zhang et al., 2009). Therefore, new therapeutic approaches for periodontal tissue regeneration are required. The development of stem‐cell‐based tissue engineering has facilitated the regeneration of functional periodontal tissues (Ding et al., 2010). However, the long‐term inflammatory microenvironment can inhibit the proliferation, migration, and regenerative potential of stem cells (Pluchino et al., 2008). Previous studies suggest stem cell transplantation promotes periodontal regeneration, of which the mechanism other than direct differentiation remains unclear. Therefore, the activation of the regenerative potential of stem cells in tissues is crucial for tissue regeneration. Recent studies have shown that source cells can secrete exosomes to transport signaling molecules to regulate host cells.

**Figure 1:**
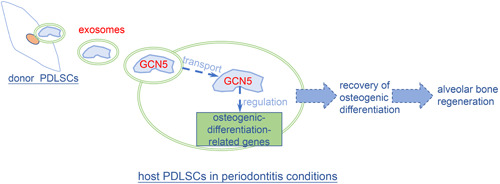
Regulatory mechanisms of Gcn5 in osteogenic differentiation of MSCs. MSCs, mesenchymal stem cells.

Mesenchymal stem cells (MSCs) are multipotent adult stem cells isolated from multiple tissues including the umbilical cord, bone marrow, and fat tissue using standardized criteria proposed by the International Society for Cellular Therapy (ISCT). MSCs maintain plastic adherence when kept under standard culture conditions and express certain markers (Dominici et al., 2006). MSCs have a high capacity for self‐renew by dividing while exhibiting great potential to differentiate into a variety of cell types such as osteocytes, chondrocytes, adipocytes, myocytes, and cardiomyocytes, making them an appealing and promising candidate for cell‐based therapeutic applications in regenerative medicine and tissue engineering, particularly in bone tissue regeneration (Bianco, 2014; Chamberlain et al., 2007). From self‐renew to oriented differentiation, chromatin is remodeled into heritable states that allow activation or maintain the repression of regulatory genes, which means specific genes in self‐renew are switched off and lineage‐specific genes in oriented differentiation are activated in response to environmental stimuli. Progression from MSCs into different differentiated lineages requires long‐lasting changes in gene expression. Though stem cell behavior is largely mediated by DNA sequence, a great number of studies have revealed that epigenetic mechanisms, namely epigenetics, would also be fundamentally important regulatory factors in stem cell fate determination, commitment, and differentiation (Ermolaeva et al., 2018; Mortada & Mortada, 2018; Teven et al., 2011). MSCs are able to secrete a greater amount of exosomes than many other cells, suggesting that exosome‐mediated intercellular information exchange may be an important way for MSCs to exert their regulatory and therapeutic functions (Figure 1).

Epigenetics refers to changes in gene expression without the alteration of the underlying DNA nucleotide sequence, which could be inherited by offspring and play an important role in the promotion of appropriate transcriptional pathways during both embryonic development and adult tissue maintenance (Brack et al., 1978; Im & Shin, 2015; Monk, 1995). Gene expression would be regulated at the epigenetic level through modification of local chromatin configuration or nuclear architecture, thus altering the accessibility of genes to transcription factors and other modulators. Specifically, these modifications regulate gene expression would result in two different forms of chromatin: one is euchromatin which is the less condensed and more accessible form of chromatin facilitating the opening of DNA to permit gene transcription; the other one is heterochromatin which is the tightly packed form of chromatin restricting the access of transcription factors to their cognate NDA binding site to repress gene transcription (Frobel et al., 2014; Meyer et al., 2016). The dynamic balance between euchromatin and heterochromatin is regulated by several epigenetic mechanisms, including DNA methylation, histone modifications, microRNAs, and chromatin remodeling. Reportedly, DNA methylation patterns are crucial for embryonic stem cell differentiation, while histone modifications and other chromatin‐based mechanisms may serve a larger role in MSCs differentiation capacity (Boquest et al., 2006; Mortada & Mortada, 2018). Therefore, the role of epigenetics, particularly histone modifications, on MSCs biology and differentiation protocols will be described in detail in this review.

Histones, the main structural components of chromatin, are small proteins that contain numerous positively charged amino acids in their N‐terminal domain, such as lysine and arginine (Bártová et al., 2008; Cheung et al., 2000). These positively charged amino acids enable histones to tightly wrap the negatively charged double‐stranded DNA. On the other hand, they can be covalently modified to change the affinity between histones and DNA to induce gene transcription or silencing. Histone acetylation and deacetylation are the widespread and dynamic modification of chromatin structure associated with the regulation of gene expression. In histone acetylation, negatively charged acetyl groups are transferred to NH_2_‐terminal lysine residues on histone proteins (Clayton et al., 2006; Huang et al., 2015). Conversely, histone deacetylation refers to the removal of the acetyl functional group from the lysine residues. In most cases, histone acetylation enhances gene transcription while histone deacetylation represses transcription (Wang et al., 2013). Histone acetylation and deacetylation are regulated by the opposing action of histone acetyltransferases (HATs) and histone deacetylases (HDACs), respectively.

HATs can be categorized into three major families based on primary‐structure homology: the Gcn5‐related N‐acetyltransferase (GNAT) family, the MYST family (containing MOZ, Ybf2/Sas3, Sas2, and Tip60), and the p300/CBP family (Dekker & Haisma, 2009; Wapenaar & Dekker, 2016) (Table 1). Several other HAT families have been identified, but they haven't been studied extensively. It should be noted that HATs are often part of large coactivator complexes that determine their binding preferences and histone acetyltransferase activity since they don't possess DNA binding sites. Gcn5 or KAT2A, a member of the GNAT family of HATs, is the first nuclear HAT protein identified from yeast, which is a transcriptional coactivator with histone acetyltransferase activity and is conserved with regard to structure as well as its histone substrates throughout the eukaryotes (Dyda et al., 2000; Salah ud‐Din et al., 2016). Gcn5 is mainly found in two functionally distinct coactivator complexes SAGA (Spt‐Ada‐Gcn5‐acetyltransferase) and ATAC (Ada‐two‐A‐containing), which is important in transcription activation mediated by interactions with transcription activators and general transcription factors (Krebs et al., 2011; Nagy et al., 2010). Gcn5 is structurally conserved throughout evolution and typically functions in a conserved fashion through the acetylation of a conserved set of lysine residues in target proteins. Thus, Gcn5 is expected to play a distinct role in the differential expression of regulatory genes during the differentiation of MSCs. Therefore, in the present review, we'll provide a comprehensive overview of recent studies on the impact of Gcn5 on MSCs biology and differentiation protocols with a focus on periodontitis and osteocytic differentiation, offering possible directions for future research in this area and further development towards therapeutic applications.

**Table 1:** The major families of histone acetyltransferases

Family	Protein	Source	Histone specificity	Known complexes
GNAT	Gcn5	Yeast/Human	H3, H4	HAT‐B, HAT‐A3
	Hat1	Yeast/Human	H4	ADA, SAGA
	PCAF	Human	H3, H4	STAGA
	Elp3	Yeast	H3, H4	RNA polymerase II
	Hpa2	Yeast	H3, H4	CPX‐2143 Hpa2acetyltransferase
MYST	Sas2	Yeast	H4	SAS‐I
	Sas3	Yeast	H3, H4, H2A	NuA3C
	Esa1	Yeast	H4, H3, H2A	NuA4, Piccolo NuA4
	MOF	Drosophilidae	H4, H3, H2A	MSL complex
	Tip60	Human	H4, H3, H2A	Tip60 complex
	MOZ	Human	H3	MOZ complex
	MORF	Human	H4, H3, H2A	MORF complex
	HBO1	Human	H4	HBO1 complex
p300/CBP	p300	Human	H2A, H2B	Numerous
	CBP	Human	H2A, H2B	Numerous
	Rtt109	Human	H3	Rtt109‐Vps75, Rtt109‐Asf1

## 2 GCN5 AND PERIODONTITIS

Periodontitis is a type of chronic inflammatory disorder characterized by gingival inflammation and alveolar bone resorption, which can cause progressive destruction of periodontal supporting tissues and irreversible resorption of alveolar bone, thus leading to the loss of a tooth (Kinane et al., 2017). Bacterial stimuli are responsible for the main pathogenic factors of periodontitis. Under the stimulation of lipopolysaccharide (LPS), immune cells within the periodontal tissues could produce inflammatory factors, such as tumor necrosis factor‐α (TNF‐α), interleukin 1β (IL‐1β) and IL‐6, which then cause a series of inflammatory responses (Lindemann et al., 1988). Local promoting factors, like poor oral hygiene, calculus accumulation, food impaction, poor restoration stimulation, and smoking, as well as systemic promoting factors, such as diabetes, cardiovascular disease, osteoporosis, acquired immunodeficiency syndrome, and genetic diseases, could promote the development of periodontitis via increased release of inflammatory factors (Manjunath et al., 2011). Periodontal tissues are mainly composed of alveolar bone, gums, periodontal ligament, and cementum. Periodontal ligament stem cells (PDLSCs), a kind of MSCs, reside in the periodontal ligament, which plays an important role in the osteogenesis balance of periodontal tissues (Trubiani et al., 2019).

As the behavior of stem cells is influenced by the microenvironment all the time, researchers believe that alveolar bone resorption caused by periodontitis has a considerable relationship with the change of PDLSCs' osteogenic differentiation capacities (Ohlstein et al., 2004). In healthy periodontal tissues, the microenvironment could protect PDLSCs and maintain a normal balance between osteogenesis and osteoclast, and no abnormal changes in alveolar bone could be observed. However, the balance would be damaged as periodontitis occurs, resulting in decreased osteogenic differentiation capacities while enhanced osteoclastic differentiation capacities (Li et al., 2016). At present, several mechanisms are applied to explain the decreased osteogenic differentiation capacities of PDLSCs caused by periodontitis: the activation of classical Wnt/β‐catenin signaling pathway to inhibit the osteogenic differentiation capacities of PDLSCs (Liang et al., 2016), the activation of nonclassical (Wnt/Ca^2+^) signaling pathway to promote the osteogenic differentiation capacities of PDLSCs (Han et al., 2016), and the activation of a PERK signaling pathway to promote the osteogenic differentiation capacities of PDLSCs (Tan et al., 2016). The mechanism can be generally concluded as follows: the periodontal pathogens could release LPS to activate or inhibit the abovementioned pathways in PDLSCs, which then triggers the release of inflammatory factors and regulates transcription factors in the nucleus, thus affecting the expression of osteogenic‐related genes and inhibiting the osteogenic differentiation capacities of PDLSCs.

Periodontitis is a complex immune‐inflammatory response. Compared with normal PDLSCs, PDLSCs from chronic periodontitis showed a low level of osteogenic differentiation in vitro, which even maintained during cell passage and seemed to have a “memory” of the previous inflammatory environment. Therefore, some researchers thought that this phenomenon may be contributed to the changes in epigenetic modification (Li et al., 2016). In addition, Ye et al. showed that MSCs underwent epigenetic changes could induce methylation of histones on osteogenic gene promoters, which then specifically downregulated the transcription of certain osteogenic genes (Ye et al., 2012). Besides, a number of studies revealed that changes in epigenetic modifications required a long period of accumulation (Gomez et al., 2009). All these results suggest that it is appropriate to explain the decreased osteogenic differentiation capacities of PDLSCs from the perspective of epigenetics in chronic periodontitis. At present, the studies of periodontitis‐related epigenetics have been mainly focused on DNA methylation, while the role of histone acetylation played in has not been studied extensively. It's found that HADC could promote the expression of Toll‐like receptor 2/4 or protease‐activated receptor in gingival epithelial cells under the action of virulence factors of Porphyromonasgingivalis, thus activating nuclear factor‐κB (NF‐κB) signaling pathway and extracellular regulated protein kinases (ERK) signaling pathway, which can lead to periodontitis (Rao et al., 2015). The addition of HDAC inhibitors to the experimental periodontitis model of Porphyromonasgingivalis can reduce the loss of alveolar bone (Ermolaeva et al., 2018). Lindroth et al. revealed that when NF‐κB was recruited to the promoters of IL‐1, IL‐2, IL‐6, IL‐8, IL‐10, and IL‐12 genes, the H3K9 acetylation of the above genes increased, and afterward the expression of these genes upregulated (Lindroth & Park, 2013). In 2016, on the basis of endoplasmic reticulum stress, Xue et al. demonstrated that chronic periodontitis negatively affected HAT MORF, which then resulted in the activation of the PERK pathway and inhibition of the osteogenesis of PDLSCs (Xue et al., 2016). Besides, it was reported that HDAC9 can deacetylate the histones H3K14 and H4K16, and afterward inhibit the family of microRNA17‐92, leading to a decrease in the osteogenic differentiation of PDLSCs in periodontitis (Chen et al., 2017). Quantitative real‐time polymerase chain reaction (qRT‐PCR) was used by Sun et al. (2017) to evaluate the expression levels of the HAT family between normal and periodontitis‐derived PDLSCs. The results revealed that compared with normal PDLSCs, the expressions of Gcn5, MOZ, MORF, EP300, and HAT1 genes in periodontitis‐derived PDLSCs were significantly decreased. All these studies suggest that HAT has a close correlation with periodontitis.

In recent years, the function of Gcn5 involved in the relationship between the osteogenic differentiation capacities of PDLSCs and periodontitis has attracted more and more researchers' attention. HAT Gcn5 can specifically acetylate histone H3K9 and H3K14 in the nucleosome of Dick‐kopf‐related protein 1 (DKK1) gene. Li et al. showed that chronic periodontitis could inhibit the expression of Gcn5 and active Wnt/β‐catenin signaling pathway, resulting in reduced osteogenic differentiation capacities of PDLSCs (Li et al., 2016). Compared with normal PDLSCs, it's found that the expressions of Gcn5 and MORF in periodontitis‐derived PDLSCs were significantly decreased (Sun et al., 2017). When small interfering RNA was used to down‐regulate the expression of these two genes, the osteogenic differentiation capacities of PDLSCs were inhibited.

Clinically, molecular methods or drugs can be used to specifically increase the expression of Gcn5 in the periodontal tissue of patients with chronic periodontitis, thereby restoring the osteogenic differentiation ability of PDLSCs and reversing the reduction of alveolar bone mass caused by chronic periodontitis, holding promise to maintain this trait in the periodontal tissues for a long time.

## 3 GCN5, BONE METABOLISM, AND OSTEOPOROSIS

Bone formation is a complex mechanism regulated by multiple factors. In recent years, the relationship between Gcn5, bone metabolism, and the related mechanisms has also received the attention of some scholars.

Osteoporosis is a common bone metabolic disease, the root cause of which is the imbalance between bone resorption and bone formation. Disorders of the skeletal vascular system can block bone formation, delay fracture healing, and then induce or aggravate age‐related or postmenopausal osteoporosis. Jing et al. found that declining histone acetyltransferase Gcn5 represses BMSC‐mediated angiogenesis during osteoporosis (Jing et al., 2017). In the study, by screening of the histone acetyltransferase family of 13 histone acetyltransferases, Only Gcn5 was significantly decreased in BMSCs derived from the osteoporotic femur. Further analysis identified that Gcn5 plays an important role in regulating the proangiogenic potential of BMSCs. Gcn5 promoted BMSC‐mediated angiogenesis by enhancing H3K9ac levels on the promoter of Vegf. In addition, the decrease of Gcn5 in osteoporotic BMSCs led to the decline of proangiogenic capacity, and overexpression of Gcn5 enhanced the proangiogenic potency of osteoporotic BMSCs. Furthermore, recovering Gcn5 expression in vivo by lentiviral expression vector significantly attenuated the loss of angiogenesis in ovariectomized mouse femurs. In the subsequent study, researchers further investigated the possible mechanism of Gcn5 regulating osteogenic differentiation under osteoporosis (Jing et al., 2018). By screening the histone acetyltransferase family, they found that during the osteogenic differentiation of bone marrow MSCs, Gcn5 expression increased, and after osteoporosis, Gcn5 expression decreased. Further analysis showed that Gcn5 promoted the osteogenic differentiation of BMSC by increasing the acetylation of histone 3 lysine 9 on the Wnt gene promoter. Reduced Gcn5 expression inhibits Wnt signaling, leading to osteogenic defects in OVX mouse BMSCs. In addition, restoring Gcn5 levels can restore BMSC osteogenic differentiation and reduce bone loss in OVX mice.

Transforming growth factor‐β (TGF‐β) superfamily are multifunctional proteins that regulate various cellular responses, including cell proliferation, differentiation, migration, and apoptosis. Kaoru Kahata et al. (2004) found that Gcn5 binds to TGF‐β‐specific R‐Smads, and enhances transcriptional activity induced by TGF‐β. In addition, Gcn5 interacts with R‐Smads for bone morphogenetic protein (BMP) signaling pathways and enhances BMP‐induced transcriptional activity. Endogenous Gcn5 is required for TGF‐β signaling and RNA interference of Gcn5 results in repression of transcriptional activities induced by TGF‐β. Therefore, Gcn5 is a Smad‐binding transcriptional coactivator that positively regulates both TGF‐β and BMP signaling pathways.

Gcn5 is an essential cofactor of COMMD1 (COM DOMAIN‐continental proton 1) ubiquitin ligase. It can mediate the ubiquitination degradation of the p65 subunit in the cell nucleus in a way that does not depend on HAT activity, thus antagonizing the NF‐kB signal and promoting the expression of the Runx2 gene and osteogenic differentiation of BMSCs (Mao et al., 2009). GCN5 has been confirmed to promote the osteogenic differentiation of BMSCs through multiple signaling pathways, and also can be regulated by mechanical signals to affect the activity of the Wnt/β‐catenin signaling pathway (Chen et al., 2017; Sun et al., 2017; Xue et al., 2016). In another study by Zhang et al., by experiments of OVXed and aged mouse models, they also found that the metabolic bone disease osteoporosis was associated with abnormal expression of Gcn5 (Zhang et al., 2016). In addition, the study revealed that regulation of osteogenic differentiation by Gcn5 is NF‐kB–dependent. NF‐kB plays an important role in bone remodeling and bone homeostasis by controlling the differentiation of bone progenitor cells. Selective inhibition of NF‐kB has been shown to block RANKL‐induced osteoclastogenesis in vivo and in vitro and prevent inflammatory bone destruction in vivo. Therefore, targeting NF‐kB may promote bone formation and inhibit bone resorption, and factors that affect its expression or transcriptional activity may be potential targets for regulating osteogenic differentiation. In the study, Gcn5 was found to inhibit NF‐kB signaling in MSCs, and most importantly, the HAT activity of Gcn5 was determined to be not required for this process.

Gcn5 is highly expressed in mouse embryos and is involved in embryonic development. In Sen et al's. (2018) study, craniofacial cartilage ossification occurs in embryonic Gcn5‐deficient mice. Under inflammatory conditions, the classic Wnt signaling pathway can exhibit completely opposite osteogenic differentiation in stem cells from different tissues. Periodontal ligament stem cells (PDLSC) in patients with periodontitis show poor osteogenic differentiation. However, the mechanism of osteogenic differentiation of PDLSCs damaged in the inflammatory microenvironment remains unclear. In Li et al's. (2016) study, they found that inflammation in the microenvironment resulted in the downregulation of histone acetyltransferaseGcn5 expression, and lack of Gcn5 resulted in decreased osteogenic differentiation of PDLSCs. The study revealed that knocking down Gcn5 reduces the expression of DKK1, which is an inhibitor of the Wnt/β‐catenin pathway, thus activating the Wnt/β‐catenin pathway of PDLSCs. Mechanistically, Gcn5 regulates the expression of DKK1 by acetylating histone H3 lysine 9 (H3K9) and histone H3 lysine 14 (H3K14) in its promoter region. Interestingly, in vivo injection of aspirin can save rat periodontitis by inhibiting inflammation and upregulating Gcn5 expression. In addition, aspirin treatment of PDLSCs upregulates Gcn5 expression and increases the osteogenic differentiation of PDLSCs. In conclusion, Gcn5 plays a protective role in periodontitis through acetylation of DKK1. The application of drugs targeting Gcn5, such as aspirin, maybe a new method for the treatment of periodontitis.

**Figure 2:**
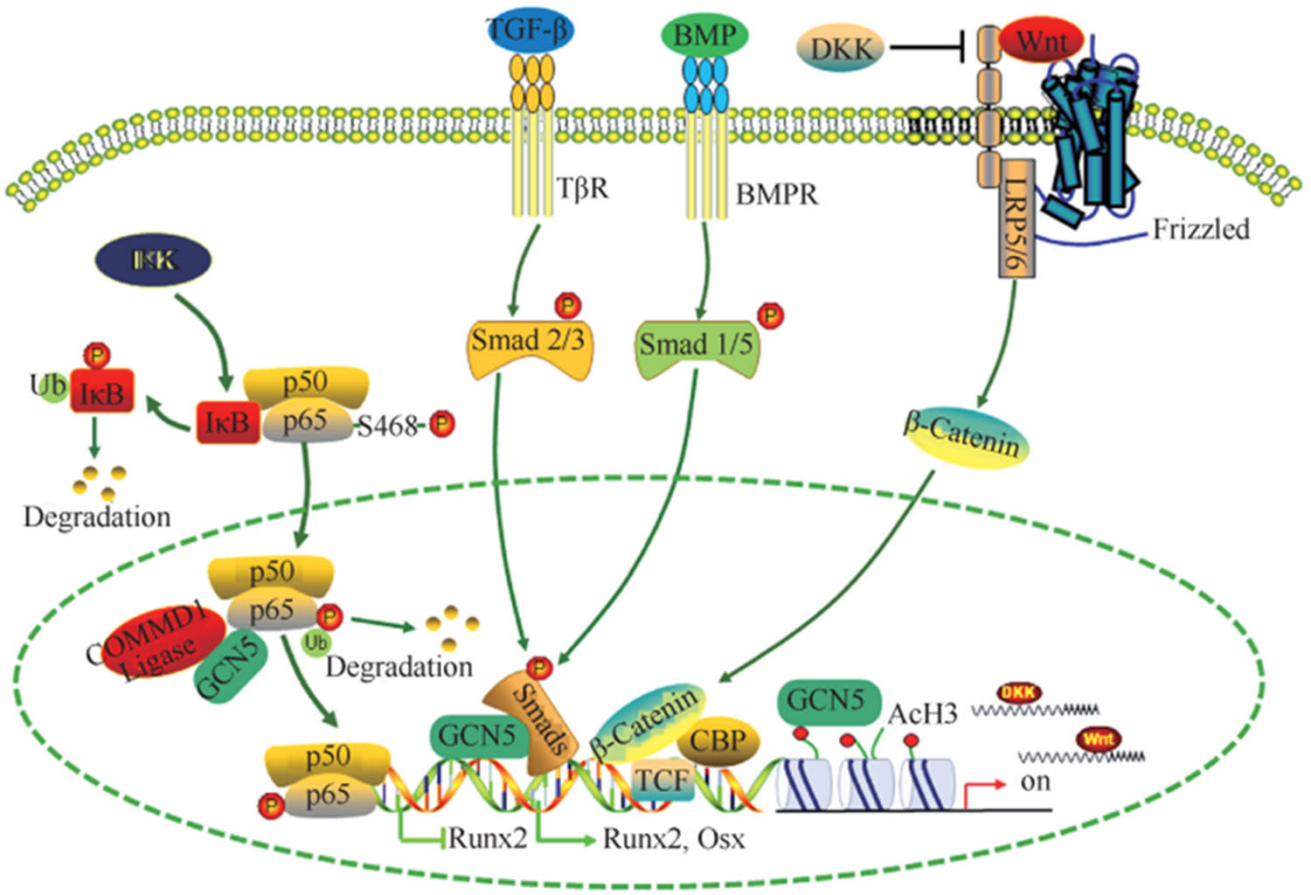
Regulatory mechanisms of Gcn5 in osteogenic differentiation of MSCs. Gcn5, general control non‐repressed protein5; MSCs, mesenchymal stem cells.

The above research suggests that Gcn5‐mediated histone modification and Wnt/β‐catenin and NF‐kB pathways play an important role in regulating cartilage development, bone marrow stem cell osteogenic differentiation and angiogenesis, and can provide new options for the treatment of bone differentiation and osteoporosis (Kahata et al., 2004) (Figure 2).

## 4 CONCLUSIONS

Protein acetylation modification plays an important role in regulating cellular chromatin remodeling and transcription factor activity. GCN5 affects the function of cell metabolism‐related genes by regulating the acetylation status of histones or non‐histones, thereby regulating some important progress of MSCs such as PDLSCs' osteogenic differentiation and BMCS osteogenic differentiation. At present, the biological effects of GCN5 in the progress have not been fully elucidated, and the design and development of its inhibitors lag far behind. Therefore, reexamining GCN5's metabolic regulation mechanism and further clarifying its mechanism of action under different physiological and pathological conditions will provide new ideas for the prevention and treatment of metabolic diseases.

## AUTHOR CONTRIBUTIONS

Wei Lu, Li Zhang, and Kun Ji wrote the manuscript. Ling Ding produced the figures. All the authors read and approved the final manuscript. Wei Lu and Li Zhang and Kun Ji contribute equally to this work.

## CONFLICT OF INTEREST

The authors declare no conflict of interest.

## Data Availability

Data available on request from the authors.
